# SURgical vs. PERcutaneous ACCESS in Transfemoral Transcatheter Aortic Valve Implantation (SU-PER-ACCESS Study)

**DOI:** 10.3390/jcm13154471

**Published:** 2024-07-30

**Authors:** Antonio Giovanni Cammardella, Marco Russo, Michele Di Mauro, Claudia Romagnoni, Fabrizio Ceresa, Francesco Patanè, Guido Gelpi, Francesco Pollari, Fabio Barili, Alessandro Parolari, Federico Ranocchi

**Affiliations:** 1 Department of Cardiac Surgery and Heart Transplantation, San Camillo Forlanini Hospital, 00152 Rome, Italy; 2 Department of Cardiothoracic Surgery, Heart and Vascular Centre, Maastricht University Medical Centre, 6229 HX Maastricht, The Netherlands; 3 Department of Cardiothoracic Surgery, IRCCS Cà Granda Ospedale Maggiore Policlinico di Milano, 20122 Milano, Italy; 4 Department of Cardiothoracic Surgery, Azienda Ospedaliera Papardo, 98158 Messina, Italy; 5 Cardiac Surgery, Klinikum Nürnberg-Paracelsus Medical University, 90471 Nuremberg, Germany; 6 Department of Biomedical and Clinical Sciences, Universitá degli Studi di Milano, 20122 Milan, Italy; 7 University Cardiac Surgery Unit, IRCCS Ospedale Galeazzi Sant’Ambrogio, 20157 Milan, Italy; 8 Harvard TH Chan School of Public Health, Boston, MA 02115, USA; 9 University Unit of Cardiac Surgery, IRCCS Policlinico S. Donato, S. Donato Milanese, 20097 Milan, Italy; 10 Department of Biomedical Sciences, University of Milano, 20174 Milan, Italy

**Keywords:** TAVI, transfemoral, vascular access, vascular closure devices (VCD), vascular complications

## Abstract

**Background:** The transfemoral (TF) approach is the most common route in TAVI, but it is still associated with a risk of bleeding and vascular complications. The aim of this study was to compare the clinical outcomes between surgical cut-down (SC) and percutaneous (PC) approach. (2) **Methods:** Between January 2018 and June 2022, 774 patients underwent a transfemoral TAVI procedure. After propensity matching, 323 patients underwent TAVI in each group. (3) **Results:** In the matched population, 15 patients (4.6%) in the SC group vs. 34 patients in the PC group (11%) experienced minor vascular complications (*p* = 0.02), while no difference for major vascular complication (1.5% vs. 1.9%) were reported. The rate of minor bleeding events was higher in the percutaneous group (11% vs. 3.1%, *p* <.001). The SC group experienced a higher rate of non-vascular-related access complications (minor 8% vs. 1.2%; major 2.2% vs. 1.2%; *p* < 0.001). (4) **Conclusions:** SC for TF-TAVI did not alter the mortality rate at 30 days and was associated with reduced minor vascular complication and bleeding. PC showed a lower rate of non-vascular-related access complications and a lower length of stay. The specific approach should be tailored to the patient’s clinical characteristics.

## 1. Introduction

Transfemoral transcatheter aortic valve implantation (TF-TAVI) has proven to be a safe and effective treatment for patients with severe aortic stenosis at high, intermediate, and low risk for open heart surgery [1,2,3,4]. Despite the reduction in sheath sizes and increased operator experience, the rate of vascular complications seems to be stable around 4–6%, as shown in recent literature [5,6,7,8,9].

Previous studies have shown that vascular and bleeding complications significantly impact morbidity and mortality after TF-TAVI [10,11].

Considering the expansion of TAVI towards low-risk and younger patients, particular attention should be addressed to reduce the rate of adverse events. In TF-TAVI, both the surgical cut-down (SC) and percutaneous (PC) approaches can be used. Several studies have compared outcomes between these two different approaches with controversial results [7]. The PC approach was associated with a lower rate of wound infection, bleeding complications and shorter hospital length of stay but a higher incidence of femoral artery stenosis and dissection [12,13,14].

The aim of this study is to analyze and compare the outcomes of SC and PC approaches in terms early results. The topic seems to be old and already analyzed in the initial TAVI experience but represents a point of extreme interest when dealing with younger and lower-risk patients who need optimal results.

## 2. Materials and Methods

### 2.1. Design and Data Collection

The SURgical vs. PERcutaneous ACCESS study is a multicenter retrospective study involving 3 Italian cardiac surgery units with experience in valve surgery and transcatheter procedures. The study has been approved by the local Ethical Committee and was not externally funded. Each participating center underwent ethical approval according to local criteria. The need for informed consent was waived for retrospective data collection. The study was sustained from the Italian Group for Research and Outcomes in Cardiac Surgery (GIROC) of the Italian Society for Cardiac Surgery (SICCH).

All adult patients (age > 18 years) undergoing transfemoral transcatheter aortic valve implantation (TF-TAVI) from January 2018 up to June 2022 in the participating centers were enrolled. The exclusion criteria were age <18 years and TAVI procedures not performed through a femoral access (e.g., trans-apical, subclavian or trans-aortic). All procedures were approved by the local heart team.

General data at baseline, peri-procedurally, at discharge, and at 30 days were retrospectively collected at each center by study consortium. Constant communication and periodical meetings between leading and secondary centers were carried out and supported by the Italian Society of Cardiac Surgery. Follow-up was performed by institutional database analysis or direct assessment by local investigators. Clinical follow-up data were 100% complete with information about living status (dead or alive), cause of death (cardiac or not) and reoperation.

The study population was divided in two groups according to access strategy: 420 patients were treated by a surgical approach (SC), and 354 patients by a fully percutaneous one (PC). The choice of type access (SC or PC) was established by the operating team on the basis of anatomical characteristics as vascular calibers, tortuosity and calcifications. All centers used both approaches.

Clinical outcomes and peri- and post-procedural complications were classified according to the Valve Academic Research Consortium III (VARC-3) criteria (Tables S1–S3 Supplementary Materials) [15].

### 2.2. TAVI Procedure

The aortic prostheses used in our study were the following: Edwards Sapien XT, Edwards Sapien 3, Sapien 3 Ultra (Edwards Lifesciences, Irvine, CA, USA), Medtronic CoreValve, Medtronic Evolut R and Medtronic Evolut PRO (Medtronic, Fridley, MN, USA), Acurate Neo, Acurate Neo 2 and Lotus (Boston Lifesciences, Marlborough, MA, USA), Portico and Navitor (Abbott, Santa Clara, CA, USA), Allegra (NVT GmbH, Hechingen, Germany).

Percutaneous vascular closure was performed using different devices: the Prostar XL and the Perclose Proglide (Abbott Vascular, Santa Clara, CA, USA) and the MANTA (Teleflex Inc., Wayne, PA, USA). The first two ones are percutaneous pre-suture-mediated vascular closure devices, while MANTA is based on a collagen plug.

The surgical access was performed in a standard fashion with a 30–40 mm transverse incision below the inguinal ligament and the sheath inserted thorough a 5/0 polypropilene purse-string sutures according to local attitude.

### 2.3. Procedural and Clinical End-Points

The primary endpoints were aimed at evaluating differences between the two treatment strategies regarding early results and were defined according the recently published VARC-3 criteria:

- Rate of vascular complication.
- Rate of access-related non-vascular complication.
- Rate of bleeding events according.

Secondary endpoints included the incidence of in-hospital and 30-day death and the other clinical outcomes at discharge and at 30 days.

### 2.4. Statistical Analysis

Descriptive statistical methods were applied to depict the study population at baseline. Continuous, normally distributed variables are presented as the mean ± standard deviation; skewed data are presented as the median and interquartile range (25th and 75th percentiles). Categorical variables are presented as numbers (%). Differences between groups were compared with Student’s *t*-test for normally distributed variables and the Mann–Whitney U test for nonnormally distributed variables. Categorical variables are summarized as the number and percentage of subjects in each category, and differences were compared with the Pearson chi-square test. A *p*-value < 0.05 was considered statistically significant.

The propensity score was obtained using the machine learning random forest method, and overlapping was tested with a common support plot 1:1; matching with different calipers from 0.5 to 0.65 was tested, choosing the best one (0.20) [16]. The variables included in the propensity model were age, sex, diabetes, NYHA class, hypertension, dyslipidemia, COPD, neurological deficit before surgery, previous myocardial infarction, liver disease, chronic kidney disease, previous cardiac surgery, previous pacemaker implantation and left ventricle ejection fraction. The balance of the two matched groups was tested with the standardized mean difference (SMD), which was considered optimal below 0.20. Adverse events were analyzed as proportions of the number of patients. The observed mortalities are described as rates (%). All deaths for unknown reasons were considered cardiac death for statistical purposes.

All reported *p*-values were considered statistically significant if below 0.05. R-Studio version 1.1.463 (2009–2018) was used for all statistical analyses.

## 3. Results

### 3.1. Patient Demographics

**Figure 1:**
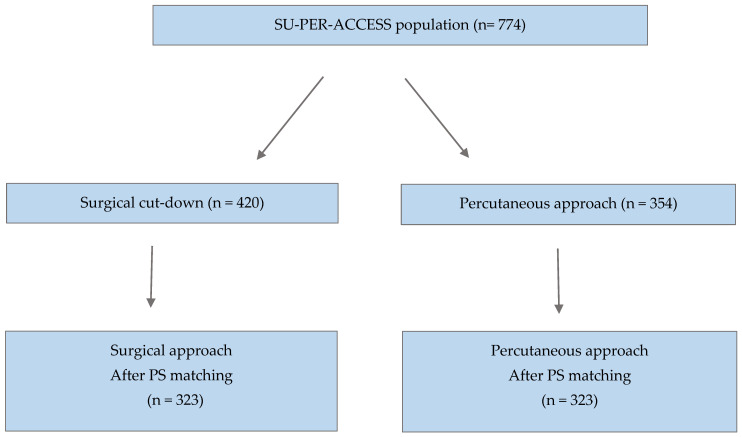
Study population. SU-PER-ACCESS: surgical vs. percutaneous access; PS: propensity score.

A total of 774 consecutive patients (mean age 81.7 ± 5.9 years; 44% female) were enrolled in the multicenter SUPER-ACCESS study at three different study sites. A surgical cut-down strategy was carried out in 420 patients (Group SURG; 54%), and the remaining 354 cases (Group PERC; 46%) were performed with a fully percutaneous approach (Figure 1). The decision to perform surgical over percutaneous TAVI was established by the operating team, according to patients’ features and anatomy (e.g., calcification at puncture site, high femoral bifurcation, vessels diameter, type of valve to be implanted) and was not protocol-related. All participating centers performed both of the procedures, and both cardiac surgeons and interventional cardiologists were included in the patient evaluation.

### 3.2. Unmatched Population

Patients in the SC (n = 420) group were more frequently male (49% vs. 39%, SMD 0.21), had more prior neurological events (11% vs. 5.1%; SMD 0.21) and were more frequently affected by diabetes (29% vs. 21%; SMD 0.18) and peripheral artery disease (19% vs. 12%; SMD = 0.21) (Table 1). The SC group was more frequently previously operated on with coronary artery bypass grafting (9% vs. 3.7%; SMD = 0.21). A valve-in-valve procedure was performed in 2.6% vs. 4.8% of cases in the SC and PC group, respectively (*p* = 0.11).

Looking at procedural features, 97% vs. 99% of cases (*p* = 0.028) of patients were treated with local anesthesia in the SC vs. PC group, whereas all types of the commercial THVs devices were used in the study groups with no differences in term of distribution (Table 2). The size of THV implanted was significantly larger in the SC group (*p* < 0.001).

### 3.3. Matched Population

After propensity matching, 323 pairs were analyzed. The two groups after matching exhibited a good balancing in term of sex, age, symptoms at baseline and distribution of pre-operative features, including neurological deficit and previous CABG. No differences in procedural data were recorded in the matched population, including prosthesis size (Table 3). This cohort represents the focus of the present analysis.

### 3.4. Perioperative Adverse Events and 30-Day Mortality

Twenty-seven patients (n = 27; 3.4%) in the entire SUPER-ACCESS cohort (n = 774) experienced death during the first 30 days. Among them, 15 were classified as cardiac related death and 12 as non-cardiac death.

No difference was recorded between the study groups for CV death (SC: 2.1%; PC: 1.7%; *p* = 0.6) and non-CV death (SC: 2.1%; PC: 0.8%; *p* = 0.16) at 30 days.

No difference in terms of 30-day mortality was confirmed after propensity match analysis (*p* = 0.19 for all-cause death).

In the matched population, 15 patients (4.6%) in the SC group vs. 34 patients in the PC group (11%) experienced minor vascular complications (*p* = 0.02), while no difference for major vascular complication (1.5% vs. 1.9%) was reported. The rate of minor bleeding events was higher in the percutaneous group (11% vs. 3.1%, *p* ≤ 0.001).

Moreover, patients treated with surgical cut-down experienced a higher rate of non-vascular-related access complications (minor 8% vs. 1.2%; major 2.2% vs. 1.2%; *p* ≤ 0.001).

The hospital length of stay was longer (*p* < 0.01) in surgical patients (median 5 days (interquartiles 4–7) vs. 4 (3–5)).

Post-operative pacemaker implantation was necessary in 7.1% of patients in the SC group vs. 3.1% in the PC group (*p* = 0.020).

No further differences in terms of other peri-operative adverse events as myocardial infarction, neurological events, pericardial effusion, need of open chest surgery or acute renal failure were described in the study cohort.

## 4. Discussion

Optimization of TAVI complication represents the main effort of current TAVI operators. Indeed, with the increasing number of procedures performed worldwide and the opening to younger and low-risk patient populations, the optimal outcome appears to be mandatory.

The percutaneous approach represents a very elegant strategy with proven safety and feasibility. Moreover, with the reduction of device’s diameter, the optimization of materials and technology used and the introduction of different device closure systems, results have improved over time. Moreover, bleeding and vascular complications remain the Achille’s heal of TF-TAVI, and the choice between percutaneous and surgical femoral access in TF-TAVI remains a subject of debate and clinical judgment. The rate of major vascular complications in TAVI is still around 4% [3,5,8,17]. A recent single-center retrospective study including 2386 patients found that the incidence of peripheral vascular intervention was 6.1%, mostly for bailout complications such as vascular stenosis/dissection or access bleeding, and that such intervention did not alter the prognosis [18]. Interestingly, even the onset of a “minor” vascular complication has been found relevant in relation to early and long-term follow-up, with 5-year survival rates being significantly lower than patients without any complications (58.0% and 70.7%, respectively) [19]. These findings, together with the expansion of the indication for TAVI, highlight the need to prevent vascular complications of all degrees.

Spot calcifications in the puncture site, vessels diameter, high femoral bifurcation and tortuosity represent risk factors for challenging the percutaneous technique. The goal of this multicenter study promoted by the Italian Society of Cardiac Surgery was to assess, in a retrospective fashion and with the use of a propensity-matched analysis, the early outcome of TAVI using surgical or fully percutaneous access. In the SUPER-ACCESS cohort, 774 cases from three Italian Cardiac Surgery Unit with experience in both PC and SC TAVI were enrolled. After matching, 324 pairs were compared. No difference in terms of mortality (cardiovascular and non-cardiovascular) was reported in the study population. Patients undergoing the PC technique exhibited a significantly higher rate (11%) of minor vascular complication vs. 4.6% in the surgical group and an increased risk of minor bleeding (11% vs. 3.1%, *p* < 0.001). No differences among major vascular and bleeding complications were detected.

Patients treated with a surgical approach presented higher rate of non-vascular-related access complications and an increased length of stay after procedure (median 5 days vs. 4).

Data from this study can be summarized as following:

- The surgical and percutaneous approaches did not differ in term of mortality.
- The percutaneous technique was associated with an increased risk of minor vascular complication and minor bleeding.
- Major vascular complications occurred with the same rate in the two groups.
- No difference in term of stroke were reported.
- Non-vascular-related access complications were associated with surgical cut-down, which increased the length of stay after the procedure.

Several studies have focused the role of different transfemoral approaches in TF-TAVI with various results and outcomes recorded. Most of the following studies refer to the first generation of devices.

Investigators from the PARTNER A and B trials analyzed the results of 857 surgical exposure TAVI vs. 559 percutaneous access cases from the initial TAVI experience. According to our data, the authors reported (in a propensity-matched analysis) similar major vascular complications in the two groups (PC 7.5% vs. SC 9.6%, *p* = 0.37), with a reduced length of stay and procedural duration in the PC group. In this study, minor vascular complications were less frequent in the PC group [20].

Data from the Spanish TAVI registry, which collected data from 2010 to 2015, suggested that PC approach was associated with a higher rate of minor vascular complications (15% vs. 4.1%). In this study, incidence of major bleeding was lower in the PC patients, and data were confirmed at 1-year follow-up [14].

In line with our data, Kochman and coauthors reported, in a large registry of 683 patients, that the rate of minor vascular complications was higher in the PC group, with no difference in major vascular complications and any bleeding [21].

A recent meta-analysis presented in 2020 and involving 5859 patients (13 studies, 1 RCT and 12 observational cohort studies) compared PC and SC access. The authors described similar rates of major vascular complications and major bleeding in the two arms, with a lower rate of minor vascular complications in the SC group but a shorter hospital stay in the PC group. These large data are in line with our series, and no difference in terms of mortality was detected [7].

Contrarily, in a propensity score-matched cohort from Kawashima et al. including patients enrolled in the OCEAN TAVI Registry from 2013 to 2015, the percutaneous approach demonstrated its superiority in terms of bleeding rate (7.2 vs. 16.9%) and major vascular complications (15 vs. 27%). In this series, the rate of overall complication was extremely high when compared with our data, possibly due to the first-generation devices used; therefore, any comparison must be carefully weighted [22].

Recently a report from a Japanese cohort of more than 300 cases focused on specific anatomic criteria which play a role in patient selection. Femoral artery depth of more than 3.5 cm sheath/femoral artery diameter ration <0.9 were independent risk factors for vascular complications and suggested parameters to select surgical cut-down. These data were missing in our series and will be assessed in prospective way [23].

Different considerations may be raised when considering the vascular closure device (VCD) used to manage large bore access.

The percutaneous vascular closure devices used in our study included Perclose Proglide and Prostar XL (Abbott Vascular) and MANTA^®^ (Teleflex Inc). The use of Proglide occurred in more than 90% of cases.

A recent meta-analysis, including seven observational studies and two randomized controlled trials and comparing MANTA with ProGlide/Prostar XL VCDs, showed similar in-hospital outcomes: risk of all bleeding, major life-threatening bleeding, major vascular complications, minor vascular complications, pseudoaneurysm, access site stenosis-dissection, blood transfusion requirement and VCD failure. In this meta-analysis, the authors found a lower device failure rate for suture-based VCD compared with Manta; however, it was not statistically significant [24].

The CHOICE-CLOSURE trial demonstrated that the use of MANTA was associated with a higher rate of access site- or access-related major and minor vascular complications compared to double-Proglide group. However, in the Proglide group, the use of a complementary device was necessary in 58.5% of patients [25].

In the present series, the VCD used did not impact the rate of vascular complications; however, as the rate of MANTA used was extremely low, and results may vary, and conclusion can be misleading.

### Limitations

This study, despite the use of the propensity score analysis, has several limitations which must be stated. First, the retrospective nature of the study conducted and the absence of monitoring may affect data quality and results. Secondly, the choice between the SC and PC approach was not per-protocol standardized but defined by each center and different operators on the basis of clinical and CT findings, with inevitable selection bias. Third, due to a lack of data collection, we did not include specific anatomical CT findings, such as calibers, vessel calcification and tortuosity or technique for percutaneous puncture as echo or fluor-guided, in the baseline characteristics. Fourth, as previously stated, the use of different THV devices and VCDs could be a confounding factor relative to the PC cohort, although the Proglide system was used in more than 90% of cases. Fifth, no long-term follow-up data are present in this study, and we cannot speculate on the role of different techniques in late outcomes. Sixth, data regarding second arterial access were missing, and therefore, we were not able to focus on this aspect.

## 5. Conclusions

In conclusion, we must state that surgical cut-down for TF-TAVI did not alter mortality rate at 30 days and was associated with reduced minor vascular complication and bleeding. Major complications did not vary through the study groups, while non-vascular-related access complications and length of stay were increased in the surgical group. Given this evidence, the two techniques must be complementary. The specific approach should be tailored to the patient’s clinical characteristics, including CT-based anatomy, and must be part of the heart team’s discussion.

## Figures and Tables

**Table 1:** Baseline clinical characteristic (before and after matching).

	Unmatched			Matched		
Characteristic	SC N = 420	PC N = 354	SMD	SC N = 323	PC N = 323	SMD
Male	206 (49%)	137 (39%)	0.21	135 (42%)	127 (39%)	0.05
Age	82 (79–85)	82 (80–86)	−0.02	82 (80–85)	82 (80–86)	−0.01
NYHA			−0.08			0.00
I	31 (7.4%)	15 (4.2%)		19 (5.9%)	15 (4.6%)	
II	160 (38%)	124 (35%)		119 (37%)	114 (35%)	
III	185 (44%)	191 (54%)		152 (47%)	173 (54%)	
IV	44 (10%)	24 (6.8%)		33 (10%)	21 (6.5%)	
Hypertension	362 (86%)	292 (82%)	0.10	275 (85%)	265 (82%)	0.08
Dyslipidemia	185 (44%)	158 (45%)	−0.01	138 (43%)	147 (46%)	−0.06
Diabetes	122 (29%)	75 (21%)	0.18	69 (21%)	73 (23%)	−0.03
COPD	124 (30%)	75 (21%)	0.19	81 (25%)	74 (23%)	0.05
Neurological dysfunction	45 (11%)	18 (5.1%)	0.21	21 (6.5%)	18 (5.6%)	0.04
PVD	81 (19%)	41 (12%)	0.21	37 (11%)	39 (12%)	−0.02
Recent MI	10 (2.4%)	7 (2.0%)	0.03	8 (2.5%)	7 (2.2%)	0.02
Cirrhosis	4 (1.0%)	7 (2.0%)	−0.09	3 (0.9%)	5 (1.5%)	−0.06
CRF	97 (23%)	73 (21%)	0.06	63 (20%)	68 (21%)	−0.04
Dialysis	8 (1.9%)	9 (2.5%)	−0.04	7 (2.2%)	8 (2.5%)	−0.02
Redo	54 (13%)	37 (10%)	0.08	27 (8.4%)	30 (9.3%)	−0.03
LVEF	59 (54, 64)	57 (55, 63)	−0.05	60 (55, 64)	57 (55, 63)	−0.02
Prior CABG	38 (9.0%)	13 (3.7%)	0.22	11 (3.4%)	13 (4.0%)	−0.03
Prior AVR	13 (3.1%)	19 (5.4%)	−0.11	13 (4.0%)	16 (5.0%)	−0.04
Prior PM	31 (7.4%)	29 (8.2%)	−0.03	19 (5.9%)	22 (6.8%)	−0.04

NYHA: New York Heart Association; COPD: chronic obstructive pulmonary disease; PVD: peripheral vascular disease; MI: myocardial infarction; CRF: chronic renal failure; Redo: previous cardiac surgery; CABG: coronary artery bypass graft surgery; AVR: aortic valve replacement; PM: pacemaker.

**Table 2:** Procedural data (before matching).

Characteristic	SC N = 420	PC N = 354	*p*-Value
Local Anesthesia	404 (97%)	351 (99%)	0.028
Valve in valve procedure	11 (2.6%)	17 (4.8%)	0.11
Pre-Implant Valvuloplasty	101 (24%)	110 (31%)	0.029
Prosthesis Size			<0.001
20	9 (2.2%)	7 (2.2%)	
23	81 (20%)	115 (35%)	
25	4 (1.0%)	0 (0%)	
26	134 (33%)	106 (33%)	
27	0 (0%)	10 (3.1%)	
29	129 (32%)	70 (22%)	
34	45 (11%)	16 (4.9%)	
Device Success	400 (95%)	334 (94%)	0.6
Technical Success	404 (96%)	335 (95%)	0.3
Cardiac tamponade	12 (2.9%)	16 (4.5%)	0.2
Emergency Cardiac Surgery	7 (1.7%)	15 (4.2%)	0.032
ICU stay (days)	0 (0–1)	0 (0–0)	<0.001
Hospital Stay (days)	5 (4, 7)	4 (3, 5)	<0.001
Vascular complications			0.013
Minor	21 (5.0%)	37 (10%)	
Major	6 (1.4%)	7 (2.0%)	
Non-vascular complications, access-related			<0.001
Minor	31 (7.4%)	4 (1.1%)	
Major	9 (2.1%)	5 (1.4%)	
Bleeding			<0.001
Minor	17 (4.0%)	36 (10%)	
Major	16 (3.8%)	5 (1.4%)	
Neurological complications			>0.9
TIA	6 (1.4%)	4 (1.1%)	
Stroke	5 (1.2%)	5 (1.4%)	
Atrial Fibrillation	31 (7.4%)	10 (2.8%)	0.005
Pacemaker implantation	46 (11%)	27 (7.6%)	0.11
Acute kidney injury	7 (1.7%)	6 (1.7%)	>0.9
Myocardial infarction	0 (0%)	0 (0%)	
Death CV-related	10 (2.4%)	6 (1.7%)	0.5
Death not CV-related	8 (1.9%)	3 (0.8%)	0.2

ICU: intensive care unit; TIA: transient ischemic attack; CV: cardiovascular.

**Table 3:** Procedural data (after matching).

Characteristic	SC N = 323	PC N = 323	*p*-Value
Local Anesthesia	314 (98%)	320 (99%)	0.13
Valve in valve procedure	11 (3.4%)	14 (4.3%)	0.5
Pre-implant Valvuloplasty	82 (25%)	103 (32%)	0.068
Prosthesis Size			
20	9 (2.9%)	6 (2.0%)	
23	70 (23%)	108 (36%)	
25	4 (1.3%)	0 (0%)	
26	102 (33%)	97 (33%)	
27	0 (0%)	10 (3.4%)	
29	94 (30%)	62 (21%)	
34	30 (9.7%)	15 (5.0%)	
Device Success	310 (96%)	305 (94%)	0.4
Technical Success	312 (97%)	306 (95%)	0.2
Cardiac tamponade	10 (3.1%)	12 (3.7%)	0.7
Emergency Cardiac Surgery	6 (1.9%)	13 (4.0%)	0.10
ICU stay (days)	0 (0, 1)	0 (0, 0)	<0.001
Hospital Stay (days)	5 (4, 7)	4 (3, 5)	<0.001
Vascular complications			0.017
Minor	15 (4.6%)	34 (11%)	
Major	5 (1.5%)	6 (1.9%)	
Non-vascular complications, access-related			<0.001
Minor	26 (8.0%)	4 (1.2%)	
Major	7 (2.2%)	4 (1.2%)	
Bleeding			<0.001
Minor	10 (3.1%)	34 (11%)	
Major	9 (2.8%)	4 (1.2%)	
Neurological complications			0.7
TIA	3 (0.9%)	4 (1.2%)	
Stroke	3 (0.9%)	5 (1.5%)	
Atrial fibrillation			
Pacemaker implantation	23 (7.1%)	10 (3.1%)	0.020
Acute kidney injury	33 (10%)	23 (7.1%)	0.2
Myocardial Infarction	6 (1.9%)	6 (1.9%)	>0.9
Death CV-related	0 (0%)	0 (0%)	
Death not CV-related	8 (2.5%)	5 (1.5%)	0.4

ICU: intensive care unit; TIA: transient ischemic attack; CV: cardiovascular.

## Data Availability

The data presented in this study are available on request from the corresponding author (privacy).

## Supplementary Materials

The following supporting information can be downloaded at: https://www.mdpi.com/article/10.3390/jcm13154471/s1, Table S1: mortality; Table S2: Vascular and access-related complications; Table S3: Technical success and device success.
