# Supplementary material for: SURgical vs. PERcutaneous ACCESS in Transfemoral Transcatheter Aortic Valve Implantation (SU-PER-ACCESS Study)
Source: J Clin Med. 2024 Jul 30;13(15):4471. doi: 10.3390/jcm13154471 (PMC11313030; doi:10.3390/jcm13154471)
Supplement: Supplementary file 1 [file jcm-13-04471-s001.zip › jcm-3060017-supplementary.pdf]

Table S1. Mortality
**Causes of mortality**
All-cause mortality
Cardiovascular mortality<br>Death meeting one of the following criteria:<br>• Related to heart failure, cardiogenic shock, bioprosthetic valve dysfunction, myocardial infarction, stroke, thromboembolism, bleeding, tamponade, vascular complication, arrhythmia or conduction system disturbances, cardiovascular infection (e.g. mediastinitis, endocarditis), or other clear cardiovascular cause<br>• Intraprocedural death<br>• Sudden death<br>• Death of unknown cause
Valve-related mortality<br>• Death presumed to be related to bioprosthetic valve dysfunction
Non-cardiovascular mortality<br>• Death clearly related to a non-cardiovascular cause: such as respiratory failure not related to heart failure (e.g. pneumonia), renal failure, liver failure, infection (e.g. urosepsis), cancer, trauma, and suicide

Table S2. Vascular and access-related complications
**Vascular complications**
Major<br>One of the following:<br>• Aortic dissection or aortic rupture<br>• Vascular (arterial or venous) injury (perforation, rupture, dissection, stenosis, ischaemia, arterial or venous thrombosis including pulmonary embolism, arteriovenous fistula, pseudoaneurysm, haematoma, retroperitoneal haematoma, infection) or compartment syndrome resulting in death, VARC type ≥ 2 bleeding, limb or visceral ischaemia, or irreversible neurologic impairment<br>• Distal embolization (non-cerebral) from a vascular source resulting in death, amputation, limb or visceral ischaemia, or irreversible end-organ damage<br>• Unplanned endovascular or surgical intervention resulting in death, VARC type ≥ 2 bleeding, limb or visceral ischaemia, or irreversible neurologic mpairment<br>• Closure device failure resulting in death, VARC type ≥ 2 bleeding, limb or visceral ischaemia, or irreversible neurologic impairment
Minor<br>One of the following:<br>• Vascular (arterial or venous) injury (perforation, rupture, dissection, stenosis, ischaemia, arterial or venous thrombosis including pulmonary embolism,

arteriovenous fistula, pseudoaneurysm, haematoma, retroperitoneal haematoma, infection) not resulting in death, VARC type ≥ 2 bleeding, limb or visceral ischaemia, or irreversible neurologic impairment<br>• Distal embolization treated with embolectomy and/or thrombectomy, not resulting in death, amputation, limb or visceral ischaemia, or irreversible end organ damage<br>• Any unplanned endovascular or surgical intervention, ultra-sound guided compression, or thrombin injection, not resulting in death, VARC type ≥ 2 bleeding, limb or visceral ischaemia, or irreversible neurologic impairment<br>• Closure device failure not resulting in death, VARC type ≥ 2 bleeding, limb or visceral ischaemia, or irreversible neurologic impairment
**Access-related non-vascular complications**
Major<br>One of the following:<br>• Non-vascular structure, non-cardiac structure perforation, injury, or infection resulting in death, VARC type ≥ 2 bleeding, irreversible nerve injury or requiring unplanned surgery or percutaneous intervention<br>• Non-vascular access site (e.g. trans-apical left ventricular) perforation, injury, or infection resulting in death, VARC type ≥ 2 bleeding, irreversible nerve injury or requiring unplanned surgery or percutaneous intervention
Minor<br>One of the following:<br>• Non-vascular structure, non-cardiac structure perforation, injury, or infection not resulting in death, VARC type ≥ 2, irreversible nerve injury, or requiring unplanned surgery or percutaneous intervention<br>• Non-vascular access site (e.g. trans-apical left ventricular) perforation, injury, or infection not resulting in death, VARC type ≥ 2 bleeding, irreversible nerve injury or requiring unplanned surgery or percutaneous intervention

**Table S3. Technical success and device success**
**Technical success**
• Freedom from mortality<br>• Successful access, delivery of the device, and retrieval of the delivery system<br>• Correct positioning of a single prosthetic heart valve into the proper anatomical location<br>• Freedom from surgery or intervention related to the device or to a major vascular or access-related, or cardiac structural complication
**Device success**<br>• Technical success<br>• Freedom from mortality

- Freedom from surgery or intervention related to the device or to a major vascular or access-related or cardiac structural complication
- Intended performance of the valve‡ (mean gradient <20 mmHg, peak velocity <3 m/s, Doppler velocity index $0.25, and less than moderate aortic regurgitation)
